# Sympathy with Nurses

**Published:** 1887-01-29

**Authors:** 


### Words of Consolation.

[Communications for this column should be addressed, "Chaplain," care of the Editor of The Hospital, who will gratefully welcome any suggestions or inquiries from matrons, nurses, and the staff generally.]

### XVIII.—Sympathy with Nurses.

We might have continued our Articles on "Sympathy" by advocating its extension amongst hospital nurses and attendants; for these may be said to need its sweet influence, if their work is to prosper, more than any others who minister to the sick and dying. But, before we venture to address the nurses themselves, we are inclined to put in a word about the sympathy which ought to be shewn *to* them. Surely when we consider the ever-recurring difficulties, aggravations, toils, and weariness of the life they lead, we must conclude that theirs is a life of all others deserving of the utmost moral and spiritual encouragement which can possibly be given it. Nurses are expected to keep themselves in constant "touch" and sympathy with every one of their patients, and yet they often have to keep up a supply of this fuel for others without receiving a word of consolation for themselves. Let the following communication which we have received from "a Matron" speak for itself:—

"Much is done for the spiritual needs of the hospital patients, but, alas! how little for the *nurses.* I have had some years' experience in large and small hospitals as a nurse, also as matron of cottage hospitals; and not one word of spiritual advice has ever been given me. The clergy who visit the hospitals seem to take it for granted that we go to church (they never ask us), and neither the nurses nor I have ever been asked if we are communicants. I think nurses, of all people, should not be left to suffer for the want of a little help, and sympathy, and advice in their own spiritual life. Their work is often weary and heart-aching; and it frequently depends on a nurse, already fatigued mentally and physically, to speak the only possible words of hope and comfort to the dying. What I propose is the formation of a guild for nurses (and those otherwise connected with hospital work) who are members of the Church of England. Let each diocese have its own branch, holding such services, &c., as would be deemed expedient by its bishop and clergy. None but those engaged in nursing know how greatly something of this kind is needed, especially in cottage and district hospitals, which have no regular chaplain, but are dependent on neighbouring clergy."

Clergy and chaplains connected with hospitals who may chance to read this, will forgive us for asking them whether they stand rebuked by the Matron's appeal. It is natural for a chaplain to feel that his first duty lies with the patients in hospital. But he has also to consider whether he has not committed to him the spiritual charge of *all* the inmates. A ministry which includes the nurses will most certainly re-act upon the patients. At the same time, it will tend to bring the entire spiritual requirements of the hospital more within the grasp and ken of the chaplain. A better understanding of the nurses in their peculiar trials and experiences will lead to a better understanding of patients likewise.

(*To be continued.*)

